# Spatio-temporal analysis of coding and long noncoding transcripts during maize endosperm development

**DOI:** 10.1038/s41598-017-03878-4

**Published:** 2017-06-19

**Authors:** Eun-Deok Kim, Yuqing Xiong, Youngjae Pyo, Dong-Hwan Kim, Byung-Ho Kang, Sibum Sung

**Affiliations:** 1 Department of Molecular Biosciences and Institute for Cellular and Molecular Biology, University of Texas at Austin, Austin, TX 78712 USA; 2 Microbiology and Cell Science, University of Florida, Gainesville, FL 32611 USA; 3 School of Life Sciences, State Key Laboratory for Agrobiotechnology, The Chinese University of Hong Kong, Shatin, New Territories, Hong Kong China

## Abstract

The maize endosperm consists of three major compartmentalized cell types: the starchy endosperm (SE), the basal endosperm transfer cell layer (BETL), and the aleurone cell layer (AL). Differential genetic programs are activated in each cell type to construct functionally and structurally distinct cells. To compare gene expression patterns involved in maize endosperm cell differentiation, we isolated transcripts from cryo-dissected endosperm specimens enriched with BETL, AL, or SE at 8, 12, and 16 days after pollination (DAP). We performed transcriptome profiling of coding and long noncoding transcripts in the three cell types during differentiation and identified clusters of the transcripts exhibiting spatio-temporal specificities. Our analysis uncovered that the BETL at 12 DAP undergoes the most dynamic transcriptional regulation for both coding and long noncoding transcripts. In addition, our transcriptome analysis revealed spatio-temporal regulatory networks of transcription factors, imprinted genes, and loci marked with histone H3 trimethylated at lysine 27. Our study suggests that various regulatory mechanisms contribute to the genetic networks specific to the functions and structures of the cell types of the endosperm.

## Introduction

Maize (Zea mays L.) is a major source of carbohydrate, protein, fat, and other nutrients for humans and livestock through its grain and biomass^1–4^. Maize endosperm development after fertilization involves unique and well-defined spatio-temporal cellular differentiation events^5, 6^. Three distinct tissue types of the maize endosperm are the starchy endosperm (SE), the basal endosperm transfer cell layer (BETL), and the aleurone cell layer (AL)^2–4, 7, 8^. The SE makes up the bulk of the endosperm and consists of storage cells packed with starch and storage proteins^9, 10^. The BETL and AL are at the endosperm surface, and these cells form an epidermis-like layer that encloses the entire endosperm and embryo. The BETL is adjacent to the maternal pedicel tissue, serving as the main interface between the maternal and filial tissues^11, 12^. The BETL functions to mediate maternal-to-filial transport and defence against microbial attack. The primary function of the AL is to mobilize nutrients stored in the endosperm during seed germination^7, 8^. Distinct functions of the three cell types in the endosperm make it an excellent model for study of cell differentiation and gene regulation mechanisms. Also, the availability of the maize genome sequence provides an opportunity to combine the existing rich genetic resources with genomics approaches^13^.

Stable gene expression in differentiated cells is often maintained by epigenetic regulation that involves chromatin modifications. Epigenetic changes in the gene regulatory network contribute to proper differentiation and maintenance of cell identity. Given that BETL, AL, and SE are derived from a single cell type but that they express distinct sets of genes, studying chromatin modifications involved in their specialization could reveal molecular mechanisms of epigenetic regulation controlling cell differentiation in eukaryotes. In addition, there is growing awareness that eukaryotic cells produce many long noncoding RNAs (lncRNAs) that play roles in various developmental programs. Mis-regulation of lncRNAs can lead to developmental defects, suggesting their contribution to the regulation of gene expression during development^14^. lncRNAs are essential *cis* and *trans* regulators of gene activity that can function as scaffolds for chromatin-modifying complexes and precursors for regulatory RNAs. Although several lncRNAs have been known for decades, it has only been appreciated recently with the advent of high-throughput RNA sequencing (RNA-Seq) that approximately 10- to 20- fold more genomic sequence is transcribed into noncoding RNA than into protein-coding RNA^15^. Despite growing numbers of identified lncRNAs in eukaryotes, functions and underlying mechanisms of lncRNA action are still poorly understood.

Several previous studies analysed transcriptomes during maize endosperm development^16–26^. These studies focused on transcripts obtained from either dissected endosperm tissues at a certain developmental stage^10, 16, 18, 19, 22, 23, 25, 26^ or whole endosperms from different developmental stages^17, 20, 21, 24^. In this study, we identified transcripts that are differentially regulated during the endosperm development by carrying out a spatio-temporal analysis of coding and noncoding transcripts in the three endosperm cell types at three stages of their development. Our analysis indicates that the transcriptomic landscapes of BETL, AL, and SE are distinct and, within the cells of the BETL, temporal expression profiles swing more significantly that in the other cells of the endosperm. Correlative changes in histone modifications and expression of lncRNAs in the vicinity of genes exhibiting spatio-temporal patterns suggest that gene regulatory programs involving chromatin modification operate in cell-type-specific manners in the maize endosperm during its development.

## Results

### Cell-type-specific transcriptome analysis during endosperm development

**Figure 1:**
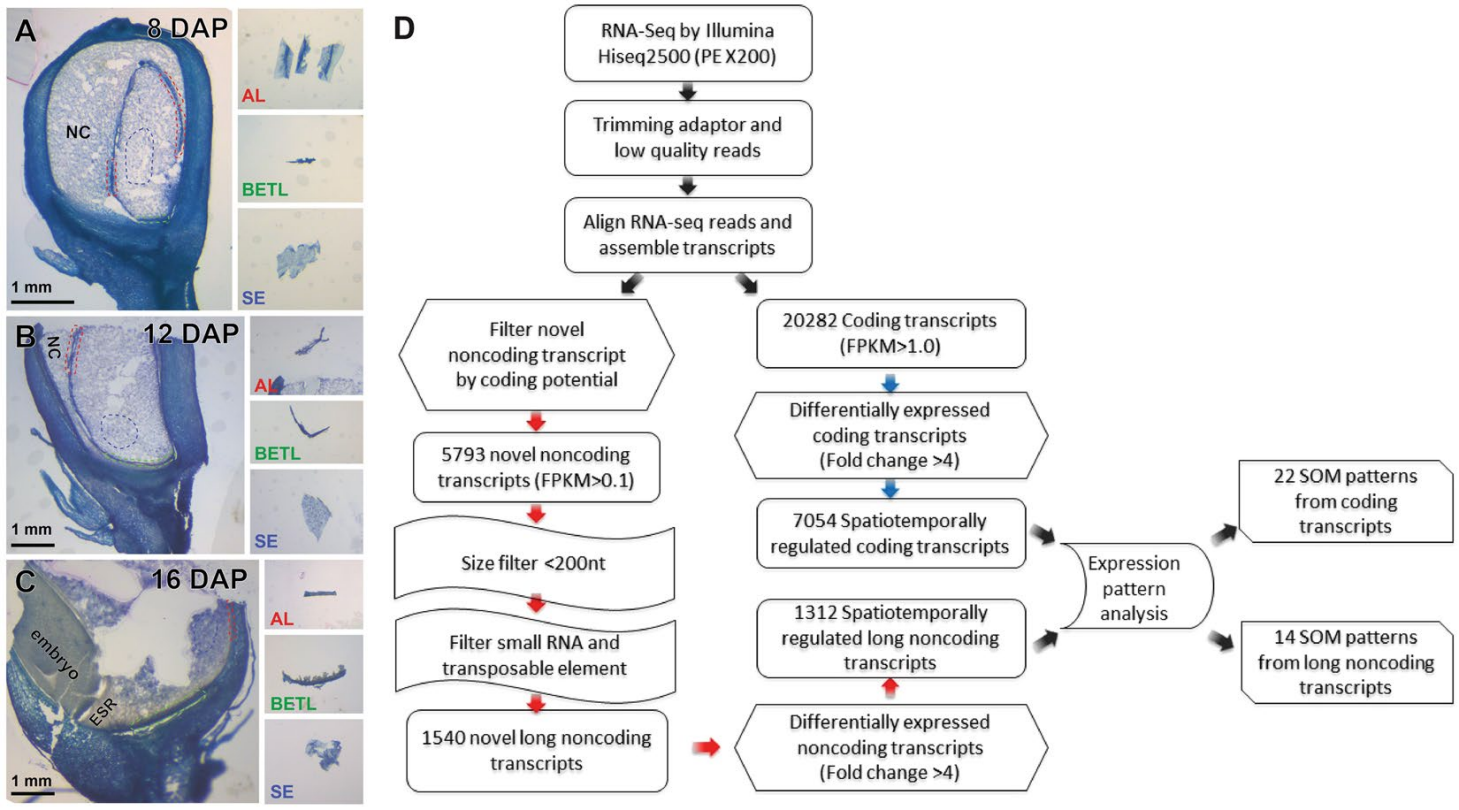
Schematic of method for transcriptome analysis of developing maize endosperm. (**A**–**C**) HistoGene-stained frozen sections from kernels at (**A**) 8 DAP, (**B**) 12 DAP, and (**C**) 16 DAP. Three insets on the right side of each panel show the three tissue types isolated from cryo-sections by free-hand dissection. The tissues surrounded by the red dashed lines and the blue dashed lines correspond to the AL and SE tissue, respectively. The BETL is marked with green dashed lines. NC, nucellus. (**D**) The bioinformatics pipeline used to identify spatio-temporally regulated coding and long noncoding transcripts at three stages of the maize endosperm development.

To address spatio-temporal transcriptional profiles over the course of endosperm development, we analysed transcriptomes of BETL, AL, and SE tissues at three different developmental stages. At 8, 12, and 16 days after pollination (DAP), fresh kernels were harvested and frozen in the field with liquid nitrogen. Because cells in the three tissues are spatially segregated in the endosperm, we were able to isolate cell specimens of BETL, AL, and SE from the cryo-sections (Fig. 1A–C). The embryo-surrounding region (ESR) is another endosperm tissue, but we did not include ESR cells in our analysis because ESR differentiation is tightly associated with the embryo development^3^. We prevented cross-contamination from ESR by isolating specimens from at least 2 mm away from the embryo. The ESR tissue is readily distinguished from BETL and SE in cryo-sections (Fig. 1C).

Poly (A)-enriched mRNA samples isolated from each cell type at each of the three different developmental stages were used to construct nine sequencing libraries. Paired-end Illumina sequencing generated an average of 20 million reads of 200-nucleotide sequence tags for subsequent RNA-Seq analysis (Table S1). The sequencing reads were aligned to the maize genome using ZmB73_RefGen_v2^13^ by Tophat^27^. More than 80% of sequence reads were mapped to the reference genome (Table S1). The aligned reads were then reassembled using the Cufflinks assembly^28^ with ZmB73_5b_filtered set, and fragments per kilobase of exon per million fragments mapped (FPKM) values were calculated for further analysis. The coding transcripts with FPKM >1.0 and the noncoding transcripts with FPKM value > 0.1 were used for further analysis (Fig. 1D).

We first examined the annotated transcript population. Coding transcripts in the combined RNA-Seq data sets represented a total of 20,282 (out of the total 39,656) annotated genes, although the total number of genes identified in each tissue type at the three developmental stages was variable (Fig. S1A). Across the three different developmental stages, the higher numbers of coding transcripts were observed in BETL samples than in the other two cell types (BETL, 17,288; AL, 16,878; and SE, 15,570; Fig. S1A). It has been speculated that transcriptional regulation in BETL and AL is most active between 8 DAP to 16 DAP due to the remarkable cell differentiation in the tissues during the period^29–31^. Although SE constitutes the majority of the endosperm by volume, SE is rather simple in its structure compared to other cell types. From 8 DAP to 16 DAP stages, SE cells mainly undergo cell proliferation^7^.

We employed several criteria to identify noncoding transcripts. First, possible partial sequence reads and artificial sequencing reads were removed based on FPKM values. Only the transcripts with an FPKM value more than 0.1 were retained. We then selected transcripts that had not been annotated in the B73 maize genome assembly^13^. The transcripts that overlapped with any known protein coding transcript were also excluded, as they may represent alternatively spliced variants. Remaining transcripts were filtered based on the coding potential using the transdecoder program^32^. A total number of 5,793 transcripts passed the criteria and were categorized as noncoding transcripts (Table S2). Similar to the distribution of the coding transcripts, noncoding transcripts were more abundant in BETL than in the other two cell types (Fig. S1B), suggesting that BETL displays wider transcriptional diversity than the other two cell types of the endosperm.

Among various noncoding transcripts, our emphasis was to catalogue novel long noncoding transcripts that may play regulatory roles in the endosperm cell differentiation. For this purpose, we applied two more filters to shortlist novel long noncoding transcripts. First, we removed transcripts shorter than 200 nucleotides. Second, we removed transcripts predicted to be small RNA precursors (such as miRNA and siRNA) or transposable elements. A total of 1,540 transcripts that satisfied all criteria were chosen for further analysis (Figs 1D and S1B).

### Identification of spatio-temporally regulated protein-coding transcripts

**Figure 2:**
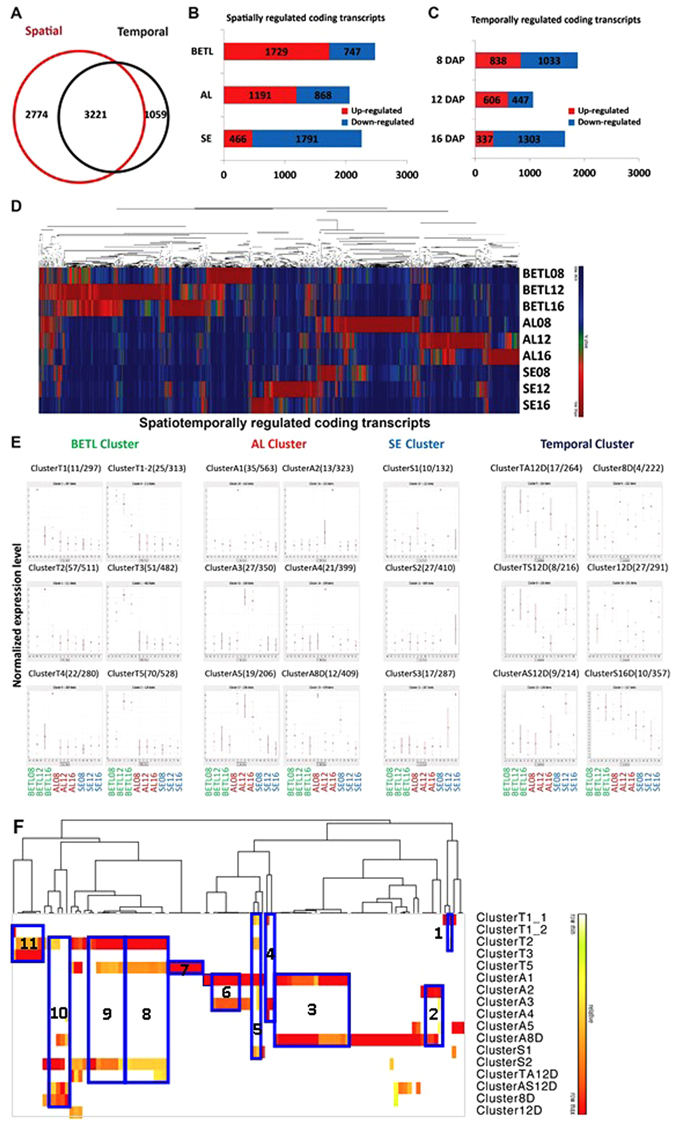
Differential expression of coding transcripts during endosperm development. (**A**) Coding transcripts with FPKM >1.0 and with more than 4-fold expression-level changes were analysed; the overlap between spatially and temporally regulated are shown. (**B**) The numbers of coding transcripts that were up- or down-regulated more than 4 fold in any tissue are shown in box graphs. (**C**) The numbers of coding transcripts that were up- or down-regulated more than 4 fold at any DAP are shown in box graphs. (**D**) Hierarchical cluster analysis of spatio-temporally regulated coding transcripts. (**E**) SOM clusters are depicted based on clustering patterns. Given in parentheses is the number of co-expressed transcription factors and the number of genes that belong to each SOM cluster. The relative level of transcript is plotted on the y axis for each of nine samples: BETL08, BETL12, BETL16, AL08, AL12, AL16, SE08, SE12, and SE16 from left to right. (**F**) Functional GO-term enrichment. Significance is represented by heat map (-log_10_ P-value; red – high/white – low). Representative functional categories correspond to numbers on graph as follows: 1, defence response; 2, oxoacid metabolic process (oxidation reduction); 3, macromolecular complex subunit organization (chromatin assembly or disassembly, nucleosome assembly, protein-DNA packing); 4, lipid biosynthesis processes; 5, response to stimulus and stress; 6, response to abiotic stimulus and biotic stress (temperature); 7, microtubule-based process; 8, post translational process (phosphorylation, protein modifications); 9, regulation of metabolic/biosynthesis process; 10, regulation of transcription process; 10, macromolecule biosynthesis process; 11, establishment of localization.

To characterize cell-type-specific patterns of expression of coding transcripts, we identified protein-coding transcripts that were highly enriched or depleted in a particular cell type. Transcripts that were up-regulated or down-regulated more than 4-fold in a single cell type are termed “spatially-regulated coding transcripts”. Similarly, coding transcripts that were either enriched or reduced at a particular developmental stage by more than 4-fold are named “temporally-regulated coding transcripts”. We identified a total number of 7,054 coding transcripts that are spatio-temporally regulated; 5,995 coding transcripts were spatially regulated and 4280 were temporally regulated (Fig. 2A). Among the spatio-temporally regulated coding transcripts, ~46% (3,221) were localized to one of the three tissue types and exhibited stage-specific expression patterns (Fig. 2A). Approximately 39% (2,774) of spatio-temporally regulated transcripts were confined to a single tissue type without stage-specific expression. Further, 1,059 (~15%) of spatio-temporally-regulated transcripts were up-regulated in a particular developmental stages but were detected in all endosperm tissue types (Fig. 2A).

Among the tissue-specific coding transcripts, 3,386 transcripts were up-regulated at one of the three-time points, and over 50% of those up-regulated genes were confined to BETL (Fig. 2B). AL and SE contained 35% and 15% of the up-regulated coding transcripts, respectively. Up-regulation patterns of coding transcripts exhibited significant shifts at each developmental stage (Fig. 2C). Transcription of protein-coding genes was most active in BETL at 8 DAP (Fig. 2D). The large number of tissue-specific coding transcripts and their oscillating patterns during maize endosperm development indicate that a transcriptional regulatory network plays a crucial role in functional differentiation of cells in the three tissues.

### Characterization of spatio-temporally regulated protein-coding transcriptome clusters

We further classified coding transcripts that exhibit spatio-temporal regulation by clustering transcripts according to spatio-temporal expression patterns to identify co-regulatory modules. Because spatio-temporal transcriptome profiles are complex and multi-dimensional, we utilized the self-organizing map (SOM) analysis tool to reveal underlying patterns. Twenty-one spatio-temporally specific clusters were uncovered by the SOM analysis (Fig. 2E). Among the 21 clusters, 15 clusters display obvious tissue type-specific patterns, including six BETL up-regulated groups, six AL up-regulated groups, and three SE up-regulated groups. Among these tissue-specific groups, ten clusters include transcripts concentrated at a single spatio-temporal point (Fig. 2E), indicating that these groups of tissue-specific genes are under tight regulation during endosperm cell differentiation.

To assign biological functions to clustered coding transcripts within distinct cell types and developmental stages, we examined Gene Ontology (GO) term enrichment using the hypergenometric distribution test and subsequent correction by the multiple hypothesis test^33^. Enriched coding transcripts with *q*-values less than 0.05 were used for the GO term enrichment analysis, and expression values of transcripts were converted into the log_10_ scale. Significantly-enriched functional categories of each cluster were tabulated and summarized by the hierarchical cluster analysis (Fig. 2F).

Functional GO-term enrichments of 17 clusters out of 21 clusters showed distinct trends. First, distinct biological functional groups are enriched in cell-type specific clusters, especially BETL and AL clusters. In BETL clusters, defence responses, proteolysis, post-translational related processes, transport, and localization groups were far more abundant than in the other two cell types throughout the three developmental stages (Fig. 2F). The lipid metabolic process and proteolysis were abundant functional groups in BETL up-regulated clusters at 8 DAP. Defense responses and stress-related groups were observed in the 8 DAP up-regulated cluster, whereas post-transcriptional regulation-related functional groups, such as protein modification and phosphorylation, are enriched in up-regulated clusters of later developmental stages (12 DAP and 16 DAP). Across all developmental stages, coding transcripts involved in intracellular transport and localization are concentrated in clusters of genes up-regulated in the BETL relative to other cell types. It has been shown that BETL accumulates anti-pathogen proteins and secretes small soluble proteins to protect maternal tissues from pathogens^7, 11, 12, 34^, thus, in this regard, BETL GO-term clusters are in agreement with previous studies.

The AL is the outermost layer of the endosperm^7, 35^. The enrichment for transcripts associated with microtubule-dependent movements and responses to abiotic stresses (Fig. 2F) is consistent with the fact that the AL differentiation involves active cell division and cell enlargement to accommodate the enlarging endosperm during development. Functional clusters of lipid biosynthesis processes are first observed in AL at 8 DAP, and their occurrences increase at 12 and 16 DAP. The oxidation-reduction and carbohydrate metabolic processes, which are related to lipid biosynthesis, were observed in AL up-regulated clusters at all developmental stages. These transcriptome signatures confirm the function of AL in which lipid bodies accumulate during seed development. In addition, AL up-regulated clusters at 8 DAP include transcription regulation via chromatin alteration and protein complex assembly (Fig. 2F), suggesting developmental reprogramming occurs at early AL differentiation.

The main function of SE is to store starch and seed proteins^7, 35^. As expected, cellular carbohydrate metabolic process, responses to abiotic stimuli and cellular polysaccharide metabolic processes were most significantly enriched GO terms in SE up-regulated coding transcript clusters.

As discussed above, the majority of distinctive GO-term functional groups in each cluster were observed among cell-type-specific SOM clusters. Some GO-term functional groups included transcripts expressed across multiple cell types, however. Enriched functional groups associated with transcriptome clusters in multiple cell types are involved in more general biological processes. For example, genes involved in cellular macromolecule metabolic processes are up-regulated in both SE- and AL-specific clusters, and enrichment in GO functional groups involved in regulation of gene expression and metabolic process was observed in both BETL- and SE-specific clusters. Carbohydrate metabolic process functional group was enriched in all tissue-specific clusters. The enriched functional groups in SE frequently overlapped with one or two tissue specific clusters. These enriched functional groups represent general metabolic and biosynthetic pathways. Taken together, distinct GO-term enrichments of spatio-temporally regulated genes indicate that coding transcripts from compartmentalized endosperm cells participate in different biological pathways that contribute to endosperm development in at distinct times and in certain regions of the organ.

### Spatio-temporally regulated transcription factor expression in developing maize endosperm

Transcription factors (TFs) play critical roles in specifying gene expression during development. Therefore, we extracted the list of TFs from the spatio-temporally regulated clusters (Figs 2E and S2). Of 2,605 annotated TFs, 492 were represented in the SOM clusters that show spatio-temporal expression patterns (Fig. 2E). All 21 clusters included sets of co-expressed TFs, indicating that various TFs show distinct expression patterns over the course of endosperm development. Of the spatio-temporally clustered TFs, 42% belong to BETL clusters, whereas AL and SE clusters account for 31% and 27%, respectively (Fig. S2A). Among 492 spatio-temporally regulated TFs, 72% were up-regulated and 28% were down-regulated in a spatio-temporal manner (Fig. S2A,B). In particular, in BETL at 8 DAP, the transcriptome includes the majority of spatio-temporal-specific TFs, suggesting that dynamic transcriptional reprogramming occurs in BETL at around this time point after pollination (Fig. S2C–E). We validated the expression patterns of selected transcription factors by qRT-PCR (Fig. S2F).

### Spatio-temporally regulated imprinted genes

Genomic imprinting is involved in plant endosperm development. Using data from several studies on parent-of-origin specific gene expression profiles in the maize endosperm^22–24, 26^, we collated a list of imprinted protein-coding genes. Subsequently, we examined whether expression of genes on this list was spatio-temporally regulated during endosperm development. Our coding transcriptome data set includes 67% (247/370) of known maternally expressed imprinted genes (MEGs) and 61% (184/298) of known paternally expressed imprinted genes (PEGs). Among sequenced imprinted genes, 75% of MEGs and 32% of PEGs belong to spatio-temporally regulated transcript clusters (Fig. S3A,B). Interestingly, both MEGs and PEGs with spatio-temporally regulated patterns were over-represented in the BETL transcriptome at 8 DAP (Fig. S3C,F). Spatio-temporally regulated MEGs also enriched in the AL transcriptome at 12 DAP (Fig. S3C,F). The majority of cell-type-specific PEGs (30/54) are expressed at the highest level in BETL, although there is no clear bias to a certain developmental stage (Figs 5F, S3D and S4B).

### Spatio-temporally regulated H3K27me3 target loci in developing maize endosperm

Chromatin-state signatures are often associated with transcriptional activity. Histone H3 trimethylation at lysine 27 (H3K27me3) is one cell-type-specific epigenetic marker in higher eukaryotes^25, 36, 37^. We examined whether H3K27me3-enriched regions corresponded to spatio-temporally regulated coding transcripts by chromatin immunoprecipitation (ChIP) followed by sequencing (Fig. S5). About 18% (306/1756) of H3K27me3-enriched regions overlapped with spatio-temporally regulated coding transcript clusters (Fig. S5A–D). Transcripts from H3K27me3-enriched loci were over-represented in BETL compared to other tissues, as BETL clusters included 45% of spatio-temporally regulated H3K27me3-marked loci (Fig. S5A). Among these temporally regulated transcripts, 44% of them were expressed at 8 DAP (Fig. S5B), suggesting the active chromatin remodelling occurs at the early developmental stage. The expression of transcripts from some of the H3K27m3-enriched loci was validated by qRT-PCR (Fig. S6).

### Identification of spatio-temporally regulated long noncoding RNA (lncRNA)

**Figure 3:**
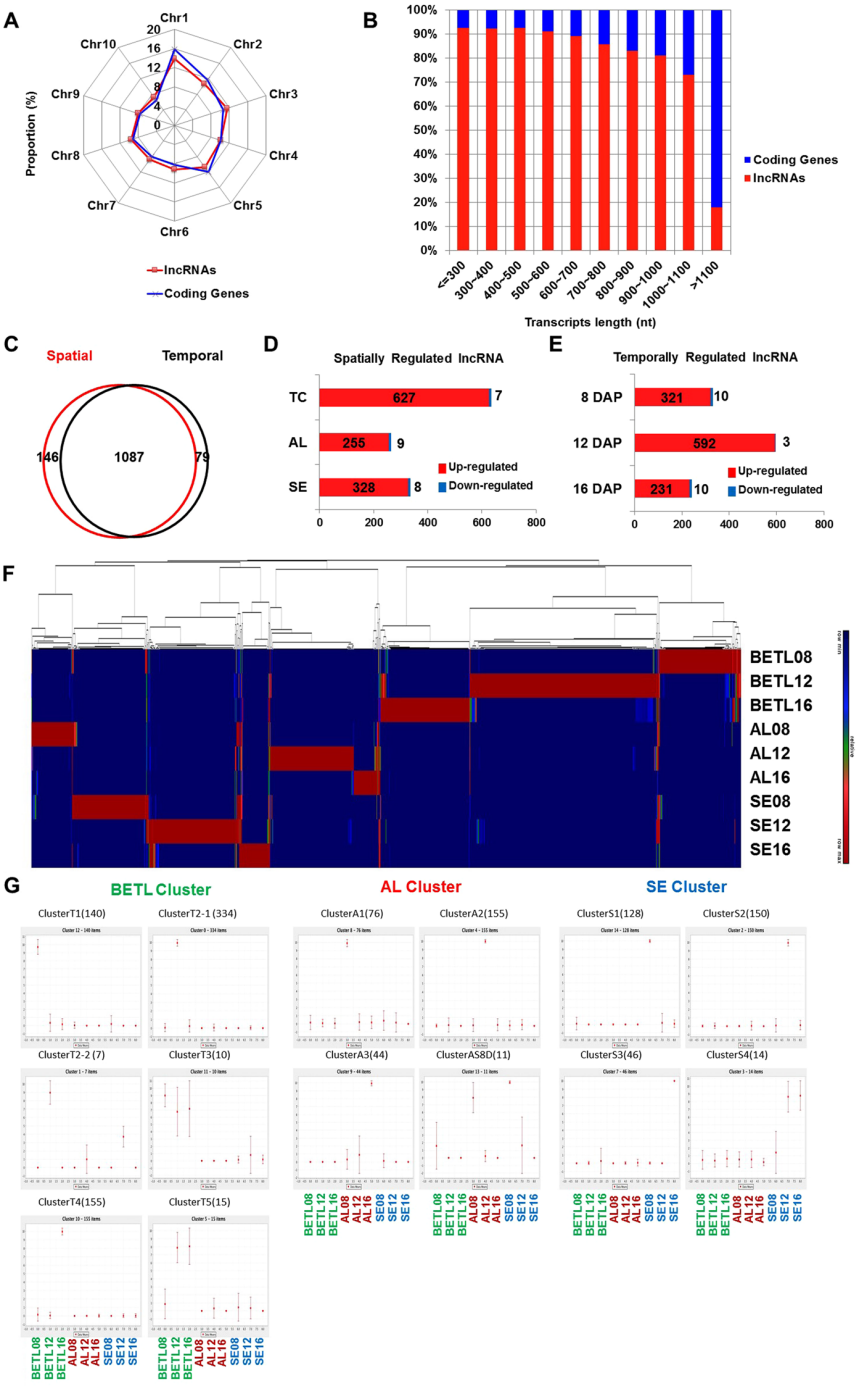
Identification of long noncoding RNAs in the maize endosperm. (**A**) Distribution of lncRNAs along each chromosome. The proportion of coding transcripts and lncRNAs for each chromosome. (**B**) Size distributions of lncRNAs and coding transcripts. (**C**) Venn diagram of the number of spatially and/or temporally regulated lncRNAs with FPKM >0.01 and more than 4-fold expression-level changes. **(D)** The numbers of lncRNAs up- or down-regulated more than 4-fold in any tissue are shown. **(E)** The numbers of lncRNAs up- or down-regulated more than 4 fold at any DAP are shown. **(F)** Hierarchical cluster analysis of spatio-temporally regulated lncRNAs. **(G)** SOM clusters depicted based on clustering patterns. In parentheses are the number of co-expressed transcription factors and the number of genes that belong to each SOM cluster. The relative level of transcript is plotted on the y axis for each of nine samples: BETL08, BETL12, BETL16, AL08, AL12, AL16, SE08, SE12, and SE16 from left to right.

As described earlier, our sequencing reads allowed us to identify a large collection of novel long noncoding RNAs (lncRNAs). lncRNAs are evenly distributed in the maize genome with the exception of slightly lower lncRNA density in chromosome 1 compared to the other chromosomes (Fig. 3A). This pattern is similar to the distribution of coding transcripts. The majority of lncRNAs are shorter in length than coding RNAs, with few (4.2%) longer than 1.1 kb. In comparison, the majority (89%) of coding transcripts were longer than 1.1 kb (Fig. 3B). To identify spatio-temporally regulated lncRNAs, we employed the same criteria used to identify spatio-temporally regulated coding transcripts (Fig. 2). Interestingly, more than 85% (1,312) of sequenced lncRNAs showed spatio-temporal expression patterns (Fig. 3C). It is noteworthy that most spatio-temporally regulated lncRNAs (over 90%) were expressed only in a certain tissue or at a certain developmental stage (Fig. 3D,E).

Among the lncRNAs, 82% were to spatio-temporally regulated, and the rest of the lncRNAs are either tissue-specific (11%) or developmental-stage specific (7%) (Fig. 3C,F). To better understand spatio-temporal patterns, an SOM analysis was used (Fig. 3G). Fourteen spatio-temporally regulated clusters were identified, with six BETL-specific clusters, four AL-specific clusters, and four SE-specific clusters. Among the spatially regulated lncRNAs, 51%, 21%, and 27% of lncRNAs were BETL-, AL-, and SE- specific lncRNAs, respectively. The spatio-temporal-specific regulation of lncRNA occurs mostly in BETL. Among temporally regulated lncRNAs, 51%, 28%, and 21% of lncRNAs were specifically expressed at 12 DAP, 8 DAP, and 16 DAP, respectively. Therefore, the BETL transcriptome at 12 DAP had the most spatio-temporally-regulated lncRNAs and coding RNAs of the endosperm tissues examined. Unlike the coding transcript cluster, however, 98% of spatio-temporally regulated lncRNAs were up-regulated only at in a certain tissue and/or at a certain developmental stage (Fig. 3D–G), indicating that the expression of lncRNAs is highly specific.

### Spatio-temporally regulation of H3K27me3 at loci encoding lncRNA

**Figure 4:**
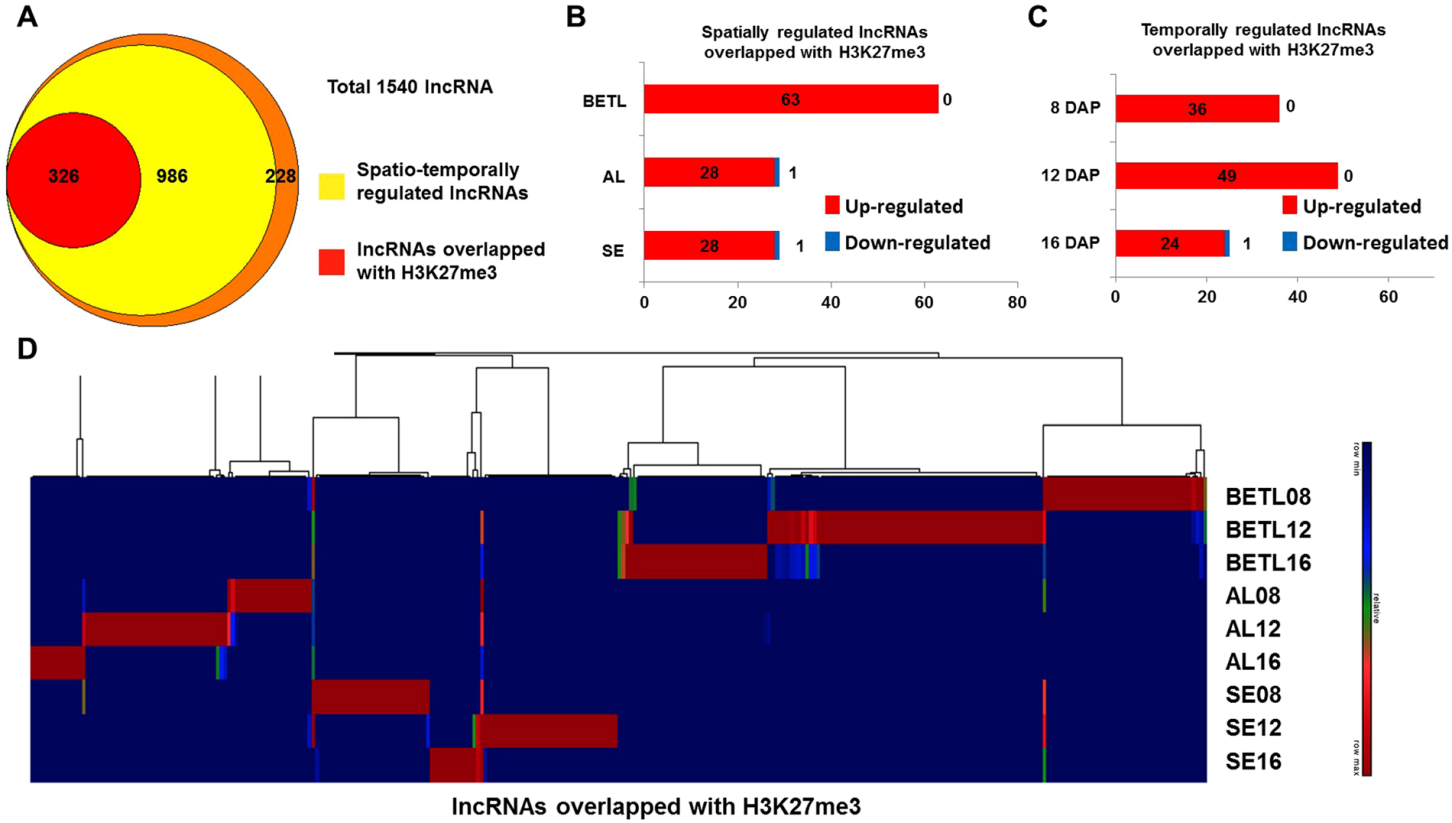
Spatio-temporally regulated lncRNA loci overlap with H3K27me3-marked loci. (**A**) Venn diagram representing the overlap between H3K27me3-enriched loci and spatio-temporally regulated lncRNA transcription sites. (**B**) The numbers of lncRNAs that overlap with H3K27me3 enriched loci that are up- or down-regulated more than 4-fold at any tissue are shown. **(C)** The numbers of lncRNAs that overlap with H3K27me3-enriched loci that were up- or down-regulated more than 4 fold at any DAP are shown. (**D**) Hierarchical cluster analysis of spatio-temporally regulated lncRNAs that overlap with H3K27me3-enriched loci.

Many lncRNAs have been implicated in mediating chromatin modification in eukaryotes^38–40^. To identify the lncRNAs that correlate with chromatin modification in endosperm cells, we compared the relative distributions of H3K27me3-enriched regions and lncRNA loci. About 30% of spatio-temporally regulated lncRNA loci overlapped with H3K27me3-enriched loci (Fig. 4A). The Chi-square test confirmed that cell-type-specific lncRNAs and developmental-stage-specific lncRNAs significantly overlapped with H3K27me3-enriched loci (Fig. S7). The majority of H3K27me3-enriched lncRNA loci were over-represented in BETL compared to the other tissues (Fig. 4B). Among developmental stage-specific H3K27me3-enriched lncRNAs, 45% of them showed 12 DAP specificity (Fig. 4C). Significant overlaps between lncRNAs and H3K27me3-enriched loci suggest that certain lncRNAs contribute to the enrichment of H3K27me3 and/or that the regulation of lncRNAs themselves are under the control of H3K27me3 (Fig. 4D). We validated the ChIP results from certain lncRNA loci by RNA *in situ* hybridization and qRT-PCR (Fig. S8).

### Spatio-temporal expression patterns of certain lncRNAs and neighbouring coding transcripts are correlated

**Figure 5:**
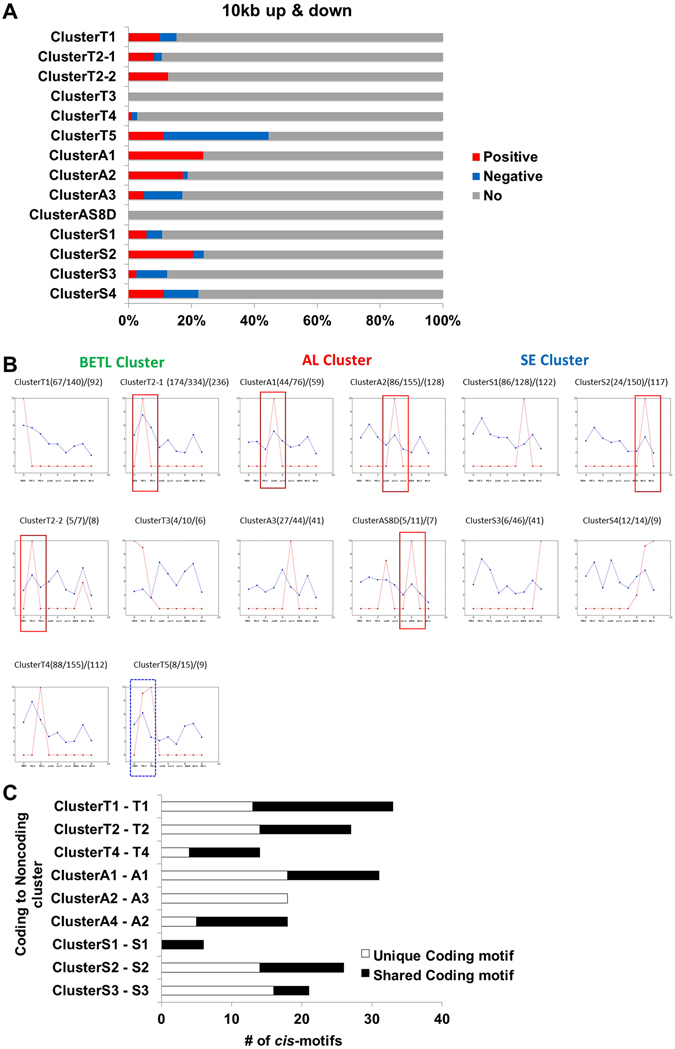
Co-regulatory relationships between noncoding and coding transcripts. (**A**) Correlations between expression of lncRNA and neighbouring coding genes within 10 kb upstream and downstream of lncRNAs transcript loci were calculated for lncRNAs in each cluster. (**B**) The median value of an expression of lncRNAs and their neighbouring coding genes in each cluster. The relative level of transcript is plotted on the y axis for each of nine samples: BETL08, BETL12, BETL16, AL08, AL12, AL16, SE08, SE12, and SE16 from left to right. Blue lines plot data for coding genes and red lines lncRNAs. Red boxes indicate positive correlations and blue box indicates negative correlation. (**C**) The number of enriched cis-regulatory modules between each SOM cluster of lncRNAs and coding genes showing similar expression patterns.

Transcription of coding genes is often regulated by neighbouring lncRNAs, and the transcription of lncRNAs can be influenced by the transcription of neighbouring coding genes^41^. To address whether the spatio-temporal expression pattern of lncRNAs is correlated with the expression of neighbouring coding genes, we compared expression patterns of each lncRNA with coding genes within 10 kb up- and downstream of lncRNA loci. In the majority of cases, there was no clear correlation between the expression of coding and noncoding transcripts (Fig. 5A), suggesting that the majority of lncRNAs loci are under their own transcriptional control.

We did observe overlap between coding and non-coding transcripts in 11 spatio-temporal expression clusters (Figs 2E and 3G). Therefore, we examined the presence of shared *cis* elements between coding and noncoding transcripts in these common spatio-temporal transcriptome clusters (Fig. 5B). We examined 5- to 22-bp sequence elements using the MEME software^42^ and the TOMTOM program^43^ to identify shared *cis*-regulatory modules with significant similarity (*q*-value < 0.05) between coding and noncoding transcripts of common spatio-temporal clusters (Fig. 5C). Nine sets of clusters exhibited significantly shared *cis* elements (Table S3). Most over-represented *cis-*regulatory motifs were found in BETL clusters of coding (38%) and noncoding (50%) transcripts. Clusters shared from 23% to 94% of total *cis*-regulatory modules. With the exception of the A3 cluster, all tissue-specific clusters of lncRNA included shared *cis*-regulatory motifs with coding genes that were clustered in similar SOM patterns. AP2/ERF binding motifs were found in all shared clusters of both coding and noncoding clusters, similar to a result using only coding transcriptome^44^. Therefore, the AP2/ERF transcription factor family may play a broad role in the transcriptional regulation of both coding and long noncoding transcripts during maize endosperm development.

### Histone modifications influence expression of spatio-temporally regulated loci

**Figure 6:**
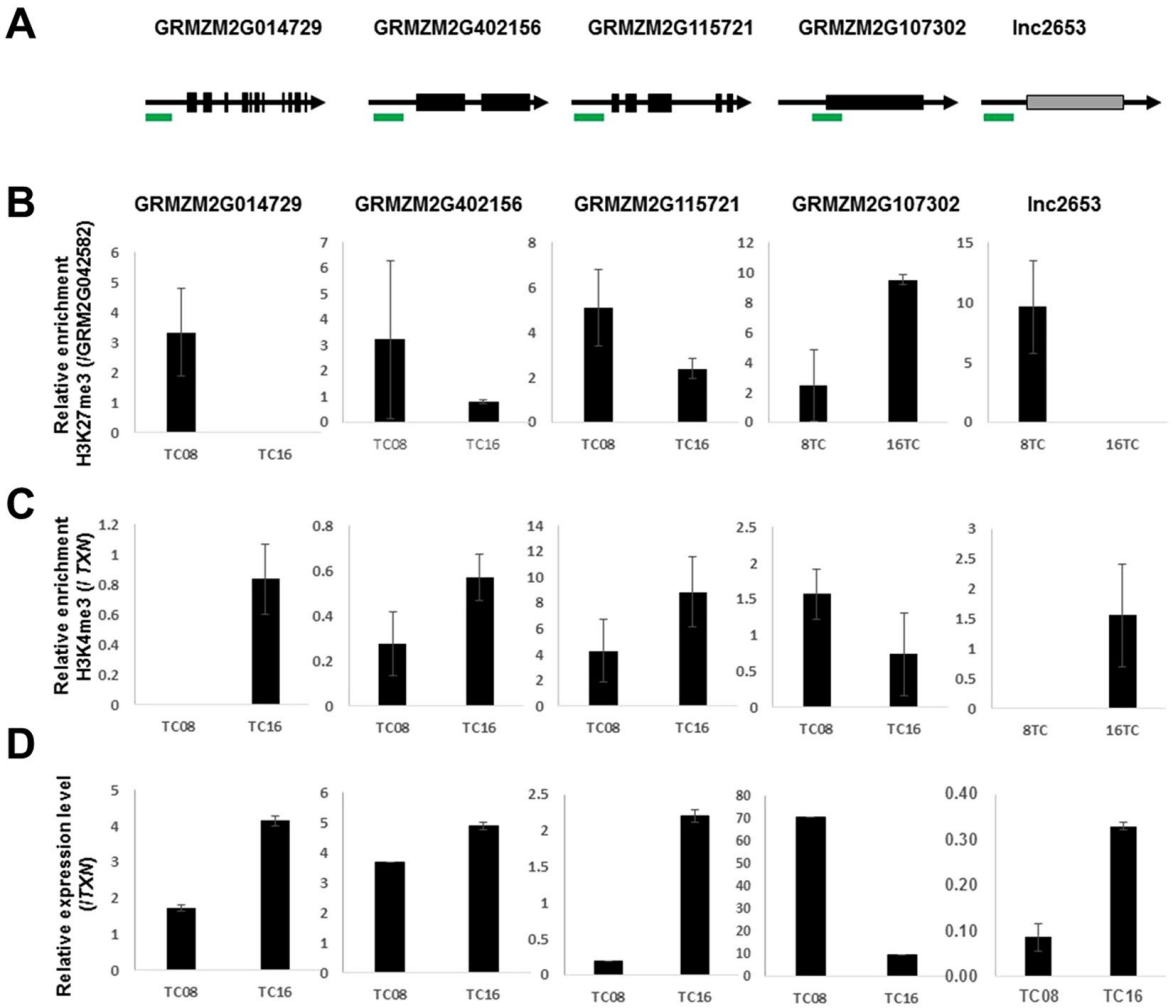
Correlation among RNA expression, H3K27me3, and H3K4me3 in BETL at 8 DAP (TC08) and 16 DAP (TC16). (**A**) Schematic representation of the locations of amplicons used for ChIP assays. (**B**) Spatio-temporal enrichment of H3K27me3 at indicated loci plotted relative to GRM2G042582. (**C**) Spatio-temporal enrichment of H3K4me3 at indicated loci plotted relative to *TXN* (GRMZM2G066612). (**D**) mRNA expression at indicate loci relative to expression of *TXN* (GRMZM2G066612).

To verify the involvement of histone modifications in the differential expression of spatio-temporally regulated transcripts, we examined several BETL-specific coding and long noncoding loci for mRNA expression, histone H3 lysine 4 trimethylation (H3K4me3) enrichment, and H3K27me3 enrichment. The differential enrichment of H3K4me3 and H3K27me3 during development has been shown in a number of developmentally regulated genes in eukaryotes, and these modifications are mediated by the Trithorax-Polycomb regulatory networks. H3K4me3 markers correlate with gene activation, whereas H3K27me3 markers correlate with gene repression^45^. As expected, we observed that the enrichment of H3K4me3 increased when transcripts were up-regulated, whereas the enrichment of H3K27me3 increased when transcripts were down-regulated (Fig. 6). Interestingly, lnc2653 also exhibited similar correlative patterns of histone modifications, suggesting that the regulation of lncRNA expression is similar to that of protein-coding transcripts and that the Trithorax-Polycomb complex mediates the spatio-temporal regulation of gene expression during endosperm development.

## Discussion

In this study, we comprehensively profiled the transcriptomes of the maize endosperm at three developmental time points in three different tissues. Previous transcriptome analyses of the maize endosperm were performed on transcripts obtained from dissected endosperm tissues at a certain developmental stage^10, 16, 18, 19, 22, 23, 25, 26^ or from whole endosperms from different developmental stages^17, 20, 21, 24^. The maize endosperm is suited for a combinatorial cell-type-specific and developmental analysis because of its compartmentalized architecture and highly differentiated nature of its cellular constituents^46^. Our study is the first to reveal the two-dimensional transcriptomic landscape of maize endosperm development.

Our analysis revealed 7,054 spatio-temporally regulated protein-coding transcripts, indicating that massive transcriptional regulations are involved in maize endosperm development. In addition, we identified 1,540 novel long noncoding transcripts and found that 1,312 were spatio-temporally regulated, suggesting that lncRNAs make important contributions to development. Using SOM analysis we classified these spatio-temporally regulated transcripts into co-expressed clusters and demonstrated that co-expressed coding and noncoding transcripts mirror the stage of endosperm differentiation within each cell type. In particular, the co-expressed clusters of coding transcripts exhibited distinct functional category enrichment patterns that are consistent with the developmental stages and the compartmentalized tissues in the endosperm. A total of 492 TFs were found in spatio-temporal clusters. These TFs may control other transcripts in the same cluster. For example, MRP-1 controls a subgroup of BETL-specific genes through a GATA-containing motif^44^. Spatio-temporally regulated TFs identified in our study are candidates for key transcriptional regulators of the endosperm development.

The abundance of genes displaying a high degree of transcriptional specificity at 8 days after pollination indicates that cell fates have been determined before 8 DAP (2). The genes that we have identified are related to maturation and maintenance of the differentiated cells as opposed to cell fate determination. Transcriptome profiling of endosperms at earlier stages is required to investigate expression of genes that determine cell fate.

In plants, imprinting is prevalent in the endosperm^47^. Previous studies have investigated imprinting in the endosperm or embryo as a whole. It is possible that mono-allelic expression can lead to the activation or repression of one allele in a cell-type-specific manner. Although our study could not examine allele-specific expression, we provide evidence that maternally and paternally expressed imprinted genes are not uniformly distributed over the endosperm. MEGs and PEGs identified from 12 DAP whole endosperm samples are available^19, 22, 23, 26^, and by comparison of our data with this previously collected data, we found that both MEGs and PEGs are most abundant in BETL clusters (Figs S3 and S4). This suggests that monoallelic regulation of either MEGs or PEGs is concentrated in the BETL. The spatio-temporal bias is more obvious in MEGs (Fig. S4), with the majority of MEG expression observed at 12 DAP in BETL (Fig. S3). The parental conflict hypothesis is a generally accepted explanation for genomic imprinting^48^. The hypothesis predicts that genes regulating growth of progeny exhibit parent-of-origin expression. *Meg1* is an example of maternally expressed imprinted gene expressed in BETL and involved in seed development^49^. Given that the rate of nutrient allocation to developing seeds is determined by maternal to filial transport through BETL, the abundance of imprinted genes in BETL agrees with the parental conflict hypothesis.

Recent studies have shown that lncRNAs play various roles in the regulation of gene expression during development in eukaryotes, from chromatin remodelling to transcriptional interference. Several lncRNAs have been shown to function in the regulation of gene expression in plants^39, 40, 50^. Other previous works examined various organ-specific transcriptomes and epigenomes^51, 52^. In our analysis, we isolated tissues by cryo-dissection and analysed lncRNA expression. Spatio-temporal expression patterns were detected for 85% of identified lncRNAs, and over 90% of lncRNAs are expressed only in a certain tissue or at a certain developmental stage (Fig. 5), supporting their roles in spatio-temporal regulation during endosperm development. Our data show that expression patterns of most lncRNAs are not correlated with those of their neighbouring genes (Fig. 5A), suggesting that lncRNA transcription is not induced by neighbouring transcriptional activity. We did, however, identify *cis* motifs that are shared among similarly clustered coding and noncoding loci (Fig. 5B,C). Therefore, lncRNAs expression appears to be transcriptionally regulated but independently of neighbouring genes. Biased expression of lncRNAs during endosperm development implies that these RNAs perform a necessary function. Interestingly, 30% of spatio-temporally regulated lncRNA loci overlap with H3K27me3-enriched loci (Fig. 4). H3K27 trimethylation is mediated by the Polycomb Repressive Complex 2 (PRC2), and our data suggest that some lncRNAs might contribute to the enrichment of H3K27me3 and/or the regulation of lncRNA itself under the control of H3K27me3. For example, we observed that the repression of lnc2653 correlates with the increased enrichment of H3K27me3 (Fig. 6).

Identification of cell-type-specific lncRNAs in our study provides evidence that lncRNAs are critical factors in differentiation of the maize endosperm. Considering that lncRNAs control chromatin structures, epigenetic mechanisms are likely to play roles in cell differentiation. It is possible to isolate chromatin specimens for ChIP experiments from the cryo-dissected endosperm tissues, as demonstrated in Fig. 6. A large-scale ChIP-Seq analysis of BETL, AL, and SE in parallel with profiling lncRNA populations is a possible direction for characterizing chromatin modification associated with cell differentiation in the plant endosperm.

## Materials and Methods

### Sample preparation and RNA analysis

All kernel samples were harvested from self-pollinated B73 ovules. Kernels were collected at 8, 12, 16 days after pollination (DAP). All collected samples were frozen immediately in liquid nitrogen and stored at −80 °C. Kernels were embedded in Tissue-Tek optimum cutting temperature-embedding compound, mounted on sectioning stub, and cut into 15–30 micrometer sections in a cryostat precooled to −22 °C (Leica Microsystems). The cryo-sectioned samples were dehydrated and stained with a HistoGene LCM Frozen Section Staining Kit according to the provided manufacturer’s protocol. Approximately 150 sections of each tissue were dissected manually under a microscope, followed by RNA extraction^12^. Total RNA was isolated using Picopure RNA isolation Kit (Arcturus) according to the manufacturer’s instructions. Genomic DNA was removed by DNase (Qiagen) treatment performed according to instructions supplied with the kit. The integrity of extracted total RNA samples was tested analysis by polyacrylamide gel electrophorese and using an Agilent 2100 Bioanalyzer. Possible contaminations were also examined by quantitative real-time polymerase chain reaction (qPCR) with standard cell-type-specific markers^12^. The RNA was further purified over a Qiagen RNeasy column. The extracted total RNA was used for relative transcript quantification by quantitative RT-PCR and high-throughput RNA-Seq analyses.

### Library preparation and sequencing

To construct RNA-Seq libraries, we first depleted samples of ribosomal RNA using the RiboMinus Plant Kit (Thermo Fisher). This is necessary to achieve optimal coverage, good detection sensitivity, and reliable results. PolyA-enriched RNA was then captured using oligo(dT) beads (Invitrogen) and was fragmented. The first-strand cDNA was synthesized by reverse transcription of the fragmented PolyA-enriched RNA, followed by second strand synthesis with DNA polymerase to generated double-stranded DNA. After end-repair and A-tailing, barcode adapters were ligated. After PCR amplification, DNA was captured on AMPure beads to purify and remove adaptor dimers. Each barcoded RNA-Seq library was sequenced on an Illumina Hiseq2500 by the University of Texas at Austin Core facility. An average of 20 million reads of 2 × 200 nucleotides paired-end sequences were generated. Prior to mapping, the sequencing reads were examined by FastQC software to remove the low-quality reads and adaptors. The remaining reads were aligned to the maize genome (ZmB73_RefGen_v2^13^ using Tophat^27^. More than ~80% of the sequencing reads from each of the nine libraries were mapped to the reference genome (Table S1). The aligned reads were then subjected to the Cufflinks assembly^28^ using ZmB73_5b_filtered set and FPKM values were calculated. The coding transcripts with FPKM value > 1 and the noncoding transcripts with FPKM value > 0.1 were used in further analyses.

### lncRNA identification

Read alignment to the reference genome was performed using the Bowtie, Tophat, and Cufflinks pipeline. The transcripts matched to annotated transcripts were filtered out using SAMtools and a customized awk script. The novel transcripts from intergenic regions were further analysed. The individual assembled novel transcripts from each library were merged into one non-redundant set using Cuffmerge option. The merged transcript assembly (final GTF file) was used in quantification of expression levels using Cufflink. Putative lncRNAs were arbitrarily defined as transcripts shorter or longer than 200 nt with lack or weak protein coding ability. The novel transcripts shorter than 200 nt were excluded using customized scripts. More than 95% of protein coding genes have open reading frames (ORFs) of more than 100 amino acids^53^. The novel long transcripts with long ORFs were removed using a coding potential program (TransDecoder tool) and customized script. To remove putative precursors of all small RNA^54^ and transposable element (TE) transcripts (http://maizetedb.org/~maize/), putative lncRNAs were aligned to small RNA and TE databases and those identical to small RNAs and TEs were removed using Python^54^ and customized scripts.

### SOM analysis

The normalized FPKM values of spatio-temporally regulated transcripts were used for self-organizing maps (SOM) analysis. SOM clustering was performed with SOM module embedded in the GenePattern based on the gene expression level (http://www.broadinstitute.org/cancer/software/genepattern). Clustering analysis was performed five times with 4,200,000 repetitions of the process, subsequently generating 21 clusters for coding transcripts and 14 clusters for lncRNAs.

### Identification of *cis*-regulatory elements

Genomic sequences were obtained from 1 kb upstream of transcription start sites of coding transcripts. For lncRNAs, the genomic sequences were extracted from 1 kb upstream of the transcription start site and 1 kb downstream of the transcription end site using customized awk scripts and Bedtools. The MEME program was used to identify *de novo* motifs in those regions. Motifs with E-value lower than 0.01 were analysed using the TOMTOM motif comparison tool. Only those with q-values lower than 0.01 were taken for further analysis to identify motifs with significantly similarity between coding RNA and lncRNA clusters.

### Chromatin immunoprecipitation and ChIP-qPCR

The cryo-sectioned tissue samples were directly cross-linked in 1% formaldehyde-containing buffer. Crosslinked tissue samples were ground in liquid nitrogen and used in ChIP experiments as previously described^55^. Antibodies recognizing H3K4me3 (Abcam cat. no.: ab8580) and H3K27me3 (Abcam cat. no.: ab6002) were used for ChIP assays. Enrichment of each fragment was first calculated based on comparison to quantitative PCR (qPCR) of input material. Second, relative enrichment was normalized by comparison to each listed reference gene (*TXN* for H3K4me3 and *GRMZM2G042582* for H3K27me3). Lastly, to show relative fold change, values of reference genes were converted to 1; relative fold changes are presented in figures. Information on primer sequences used in ChIP-qPCR analyses are shown in Supplemental Table 3. qPCR was performed using Maxima SYBR green master mix (Thermo Scientific) according to the manufacturer’s instructions. All ChIP-qPCR analyses were performed with at least two biological replicates and with two or three technical replicates. qPCR was performed in a 384-well PCR plate using the ViiA^TM^ 7 Real-Time PCR System (Life Technologies).

### Quantitative RT-PCR

First-strand cDNA synthesis was performed with 1 μg of total RNA and oligo(dT) for coding genes or random primers for noncoding RNAs using M-MLV Reverse Transcriptase (Promega). Real-time qRT-PCR was carried out using Maxima SYBR Green/ROX qPCR Master Mix (Thermo Scientific) on a ViiA7 Real-Time PCR system (Applied Biosystems). The PCR cycling conditions were as follows: 95 °C for 10 min, 40 cycles of 95 °C for 15 sec, 60 °C for 30 sec, and 72 °C for 30 sec. The relative transcript levels of coding genes and lncRNAs were determined by normalization of the resulting expression levels to that of *thioredoxin* (Zm*TXN*, gene ID GRMZM2G066612) as described^56^. The primer sequences used in qRT-PCR analysis are in Table S3. All primer pairs were selected using the Primer3 program and were tested in 3% agarose gel to ensure the specificity and efficiency of each primer set.

### *In Situ* hybridization

The template for preparing an *in situ* hybridization probe to localize lnc2653 was amplified from a maize genomic DNA sample with a primer pair: 5′- GAGCAAGTCCAGCTGTTACATATATCTTTATAGAATATATAATTATTGCTACTCTTACAT-3′ and 5′-ACTATTCGGTGACCAGCTTGAGCACGGTTGTTGTTGTATTATATTAATTGGATCTGACGCATCCC-3′. The DNA sample was cloned into the pGEM-T easy PCR cloning vector (Promega). Two types of RNA probes were prepared with DIG RNA Labeling Kit (Sigma-Aldrich) using the T7 and SP6 primers. These two RNA probes have sequences complementary to each other. Caryopses of maize 12 DAP inbred W22 were fixed, dehydrated, and embedded in Paraplast Plus paraffin (Sherwood Medical Co.) Sections were prepared from the embedded caryopses with a Leica RM2235 rotary microtome (Leica-Microsystems). Hybridization, washing, blocking, antibody labelling, and colour reaction were carried out as previously described^12, 57^.

## Electronic supplementary material

Supplementary Information
