# Supplementary material for: Spatio-temporal analysis of coding and long noncoding transcripts during maize endosperm development
Source: Sci Rep. 2017 Jun 19;7:3838. doi: 10.1038/s41598-017-03878-4 (PMC5476630; doi:10.1038/s41598-017-03878-4)
Supplement: Supplementary file 1 — Supplementary Information [file 41598_2017_3878_MOESM1_ESM.pdf]

Supplementary Information

“Spatio-temporal analysis of coding and long noncoding transcripts during maize endosperm development”

Eun-Deok Kim, Yuqing Xiong, Youngjae Pyo, Dong-Hwan Kim, Byung-Ho Kang, and Sibum Sung

Supplementary Tables S1 ~ S3
Supplementary Figures S1 ~ S8

Table S1. Summary of alignment of RNA-seq reads to maize B73 genome.

	Total Reads	Mapped Reads	Fraction Tophat(%)	Fraction BWA(%)
**AL08**	20,930,227	16,892,600	80	81
**AL12**	20,100,056	16,665,972	76	83
**AL16**	19,183,803	17,237,607	82	90
**SE08**	20,117,161	17,249,451	78	86
**SE12**	20,248,540	17,852,491	82	88
**SE16**	20,461,456	17,859,064	80	87
**BETL08**	22,881,396	19,915,567	81	87
**BETL12**	20,257,413	15,961,406	72	79
**BETL16**	18,761,116	16,415,752	80	87

ZmB73_RefGen_v2.tar.gz 2010
ZmB73_5b_FGS.gff.gz 07-Feb-2011

Table S2. Summary of identified novel transcripts.

	**Total number of Novel transcripts**	**Total number of novel noncoding transcripts**
**BETL08**	2693	1221
**BETL12**	5475	2710
**BETL16**	3752	1866
**AL08**	1435	652
**AL12**	2734	1263
**AL16**	1152	566
**SE08**	1113	992
**SE12**	3317	1519
**SE16**	1403	710

Table S3. Primer sequences used in this study

Name	Sequence		Purpose
GRMZM2G014729-real-F	GACGAGCACAACAGCAAAG		qRT-PCR
GRMZM2G014729-real-R	GCTAGGTAGAGAGTAGAGAGTAGAG		qRT-PCR
GRMZM2G402156-real-F	AGTGGAACCTACAACCCAAATC		qRT-PCR
GRMZM2G402156-real-R	CTGGTGCTCTCTTGGTCAATAC		qRT-PCR
GRMZM2G115721-real-F	GATGAGCAGCTACACCAACA		qRT-PCR
GRMZM2G115721-real-R	TGGACCTCCTCGAACTACAT		qRT-PCR
GRMZM2G107302-real-F	TCAAGTGTTCGTCGCTGTC		qRT-PCR
GRMZM2G107302-real-R	GAAACGGTTGCAGTCGTAATTC		qRT-PCR
lncRNA2653-real-F	CGCATCGCTCAGAGAAAGAA		qRT-PCR
lncRNA2653-real-R	ACTGCCAGAAGAACAACTACAA		qRT-PCR
GRMZM2G016145-real-F	TGC ACT GAT GTC ATA CCA GAA A	Orphan123	qRT-PCR
GRMZM2G016145-real-R	ATA CGA GGC ACG TCA GAA AC		qRT-PCR
GRMZM2G444748-real-F	GATGCGCCAGCATCTCTC	ZIP60	qRT-PCR
GRMZM2G444748-real-R	GGTGGCTAGCTAGTGTAGGT		qRT-PCR
GRMZM2G402156-real-F	AGTGGAACCTACAACCCAAATC	MYBR19	qRT-PCR
GRMZM2G402156-real-R	CTGGTGCTCTCTTGGTCAATAC		qRT-PCR
GRMZM2G089949-real-F	GACTTCGTACCTGAAGCCTATC	DOF6	qRT-PCR
GRMZM2G089949-real-R	GCTCTCTGTTGTCCTTTCCA		qRT-PCR
GRMZM2G104866-real-F	GGCGATAACTTCACCGACTAC	EREB203	qRT-PCR
GRMZM2G104866-real-R	CGACTTGGAGCAGCACAT		qRT-PCR
GRMZM2G041996-real-F	GGGACGACATCAAGGTCATAG		qRT-PCR
GRMZM2G041996-real-R	GTGGTGGTGGTGGTGAAG		qRT-PCR
GRMZM2G098892-real-F	AGTGAATCTGCTGAGAGAGAAATAG		qRT-PCR
GRMZM2G098892-real-R	CACGTCTGTGCTACTGATGAA		qRT-PCR
lncRNA1899-real-F	GCATCGTCTCTTTCGGTGTA		qRT-PCR
lncRNA1899-real-R	GAGCAAGACCAAGGTGAACT		qRT-PCR
lncRNA5634-real-F	GATGAACCATGATTGTGGTAATGA		qRT-PCR
lncRNA5634-real-R	GGGACATGAAACAAAGGTGAC		qRT-PCR
TXN_ChIP_F	GCCACGACTCTTCCAGTTT		ChIP-qPCR
TXN_ChIP_R	TCGGTCAGATCAACACCAATC		ChIP-qPCR
GRMZM2G042582-F	ACCATCCGATCCGGTAGTATC		ChIP-qPCR
GRMZM2G042582-R	GCTGGATTCGGTATGTGTGT		ChIP-qPCR
ZM2G014729_ProF	GCAGATTTAACGGCGGATATTT		ChIP-qPCR
ZM2G014729_ProR	TCTATCTAGTGCCCATTGAAGAAT		ChIP-qPCR
ZM2G402156_ProF	ATAAAGCACCGCAATCATGAAA		ChIP-qPCR
ZM2G402156_ProR	TGTGGACACCCACATATGTATC		ChIP-qPCR
ZM2G115721_proF 2	GGCTACTCAAAGCCAGACAT		ChIP-qPCR
ZM2G115721_proR 2	AGCCACTACACTACAAAGATACAC		ChIP-qPCR
ZM2g107302-BodyF	CGCCATCAGGACGACAG		ChIP-qPCR

ZM2g107302-BodyR	CATGTGCATGAGATAGATATATGTGG		ChIP-qPCR
CUFF.2653 Right F	ACCTTCTATCGTATCGCAAGTAAA		ChIP-qPCR
CUFF.2653 Right R	TGAAAGGGAAAGAAGTGGAGAG		ChIP-qPCR

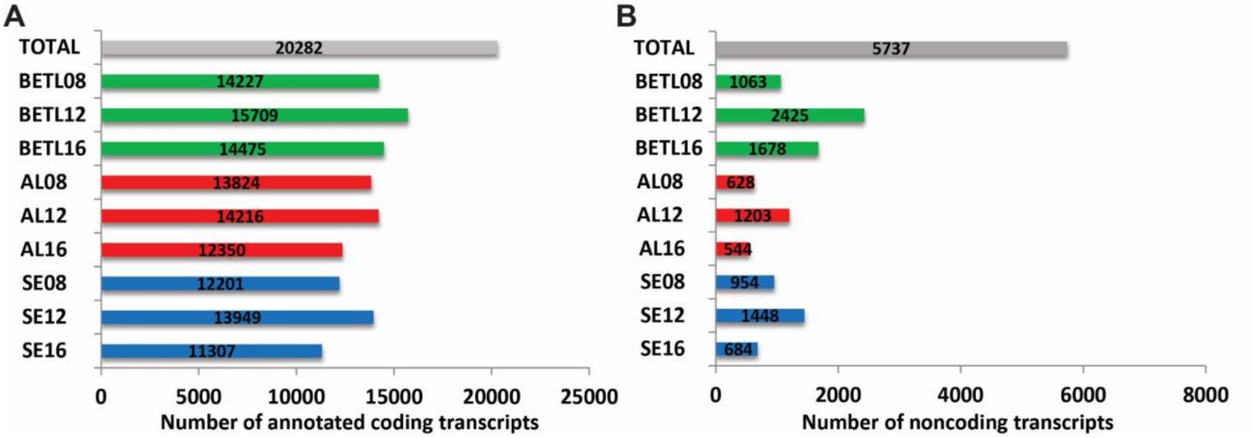

Figure S1. Transcriptome dynamics during the maize endosperm differentiation and development. **(A)** The number of annotated coding transcripts represented in each of the nine RNA-Seq data sets (FPKM>1). **(B)** The number of identified long noncoding transcripts represented in each of the nine RNA-Seq data sets.

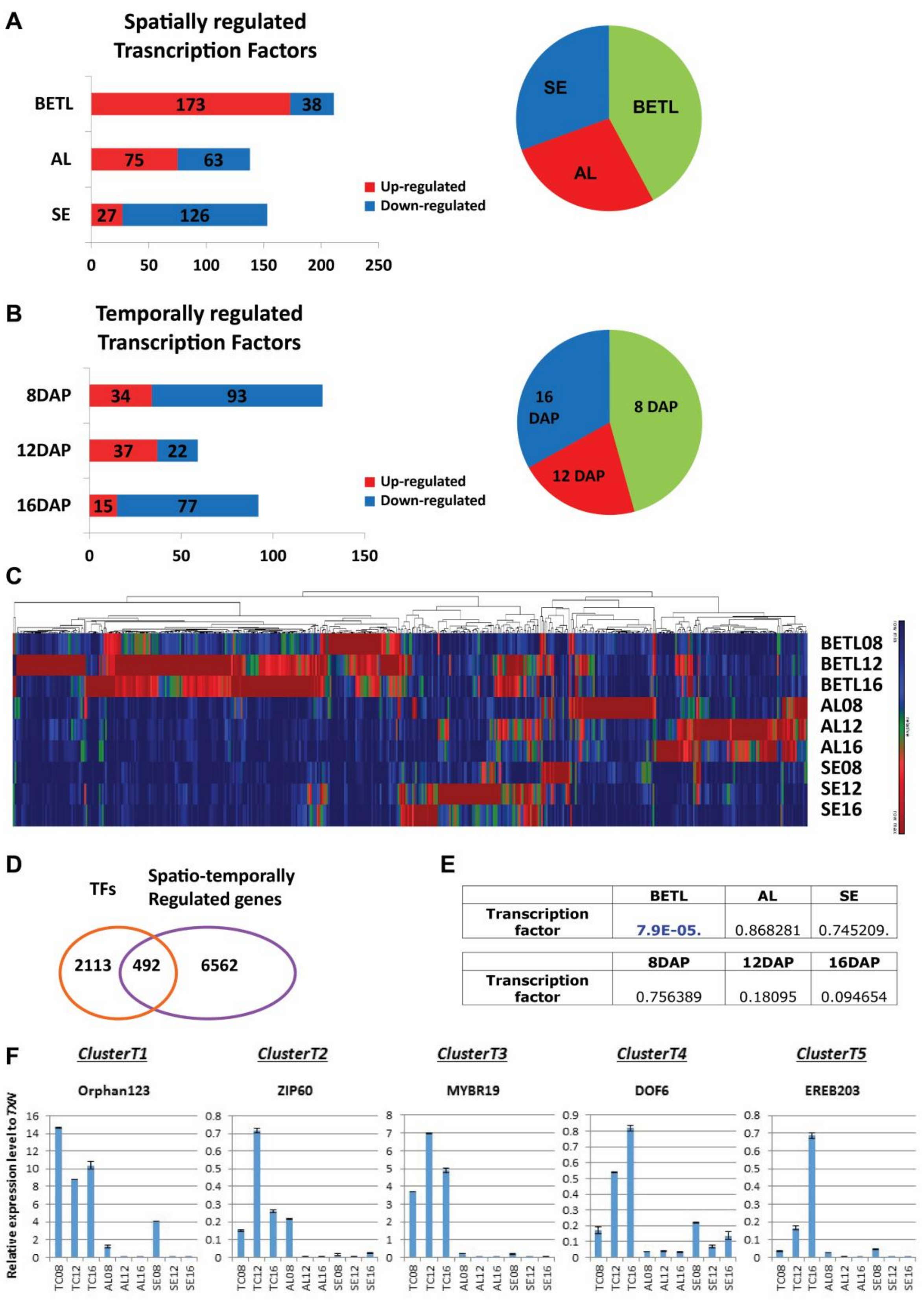

	BETL	AL	SE
Transcription factor	7.9E-05.	0.868281	0.745209.

	8DAP	12DAP	16DAP
Transcription factor	0.756389	0.18095	0.094654

Figure S2. Spatio-temporally regulated Transcription Factors. **(A)** The number of Transcription factor that are up- or down-regulated more than 4-fold at any tissue is shown in box graphs. **(B)** The number of Transcription factor that are up- or down-regulated more than 4 fold at any DAP is shown in box graphs. **(C)** Hierarchical cluster analysis of spatiotemporally regulated Transcription factors. **(D)** Venn diagram of the number of overlapping transcription factor genes and spatiotemporally regulated genes during maize endosperm development. **(E)** Statistical measure of biological significance for overlapping genomic region of transcription factor genes by Chi-square test. **(F)** Validation of the expression of spatiotemporally regulated Transcription Factors.Orphan123 (GRMZM2G016145) bZIP60 (GRMZM2G444748) MYBR19 (GRMZM2G402156) DOF6 (GRMZM2G089949), and EREB203 (GRMZM2G104866) were tested using qRT-PCR analysis. *Y*-axis represents the relative expression level to *TXN* (GRMZM2G066612) and *X*-axis indicates 9 samples: BETL08, BETL12, BETL16, AL08, AL12, AL16, SE08, SE12, and SE16 from left to right.

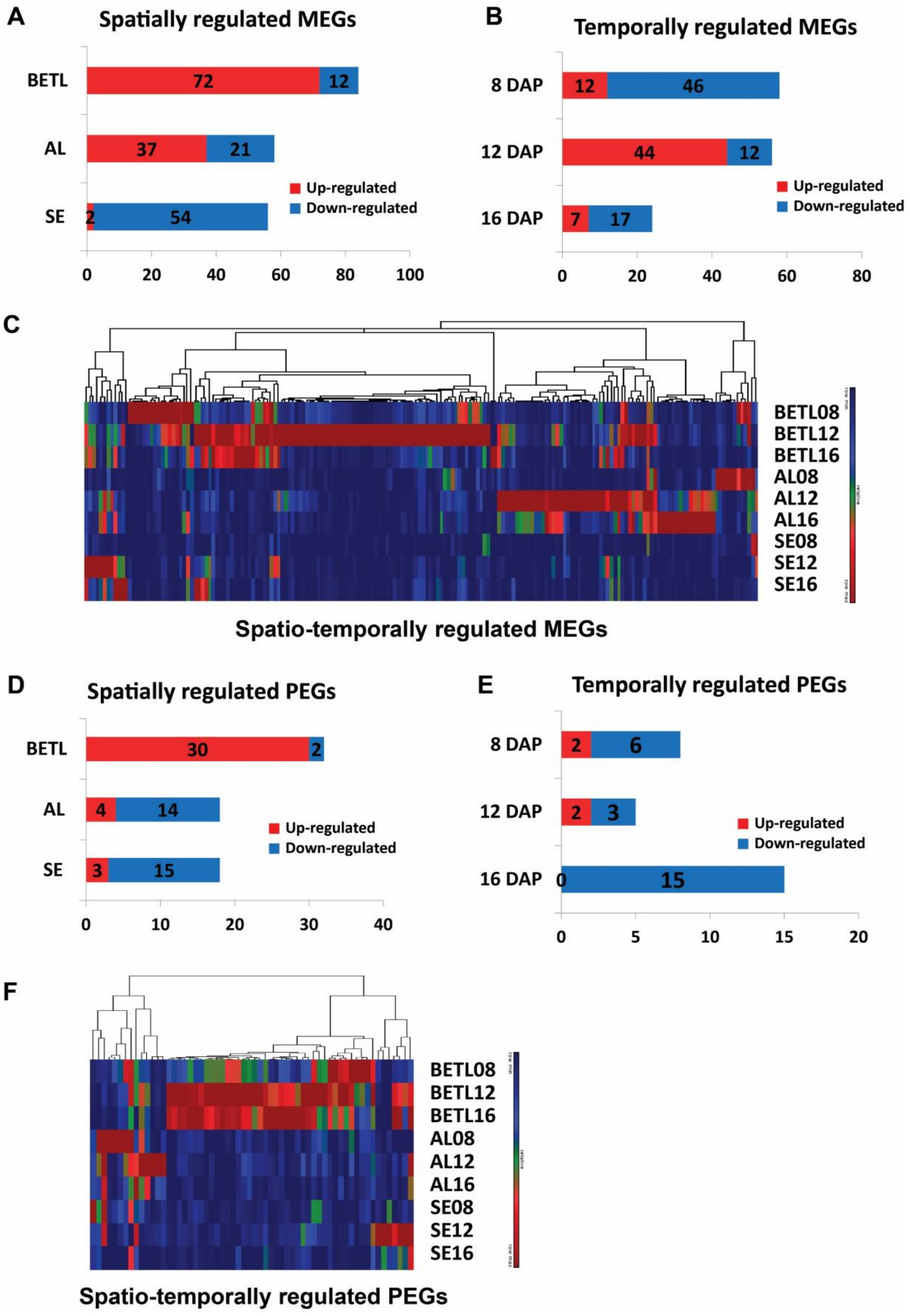
A
Spatially regulated MEGs
BETL
AL
SE
72
12
37
21
2
54
Up-regulated
Down-regulated
0
20
40
60
80
100
B
Temporally regulated MEGs
8 DAP
12 DAP
16 DAP
12
46
44
12
7
17
Up-regulated
Down-regulated
0
20
40
60
80
C
BETL08
BETL12
BETL16
AL08
AL12
AL16
SE08
SE12
SE16
Spatio-temporally regulated MEGs
D
Spatially regulated PEGs
BETL
AL
SE
30
2
4
14
3
15
Up-regulated
Down-regulated
0
10
20
30
40
E
Temporally regulated PEGs
8 DAP
12 DAP
16 DAP
2
6
2
3
0
15
Up-regulated
Down-regulated
0
5
10
15
20
F
BETL08
BETL12
BETL16
AL08
AL12
AL16
SE08
SE12
SE16
Spatio-temporally regulated PEGs

Figure S3. Spatio-temporally regulated MEGs and PEGs. **(A)** The number of MEGs that were up- or down-regulated more than 4-fold in any one tissue was shown in box graphs. **(B)** The number of MEGs that were up- or down-regulated more than 4 fold at any DAP is shown in box graphs. **(C)** Hierarchical cluster analysis of spatiotemporally regulated MEGs. **(D)** The number of PEGs that were up- or down-regulated more than 4-fold at any tissue is shown in box graphs. **(E)** The number of PEGs that were up- or down-regulated more than 4 fold at any DAP is shown in box graphs. **(F)** Hierarchical cluster analysis of spatiotemporally regulated PEGs.

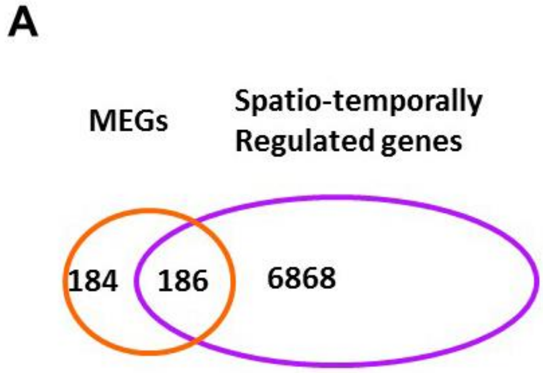

Figure S4. Significant overlapping between MEGs/PEGs and spatio-temporally regulated genes. **(A)** Venn diagram of the number of genes overlapping between MEGs and spatio-temporally regulated genes during maize endosperm development. Statistical significance were measured by Chi-square test. **(B)** Venn diagram of the number of genes overlapping between PEGs and spatio-temporally regulated genes during maize endosperm development. Statistical significance were measured by Chi-square test.

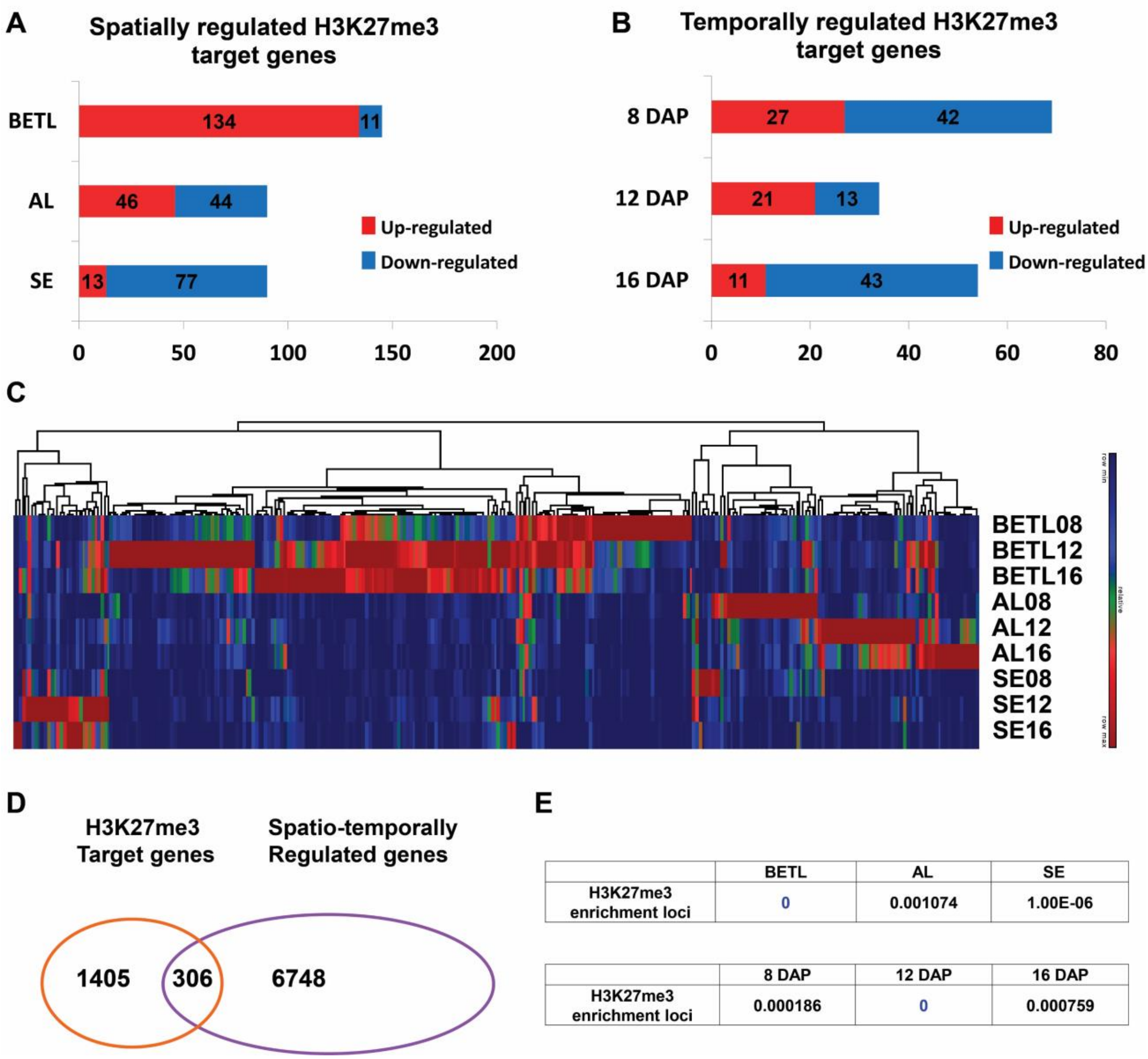

Figure S5. Spatio-temporally regulated H3K27me3 target coding loci. **(A)** The number of H3K27me3-enriched loci that were up- or down-regulated more than 4-fold at any tissue is shown in box graphs. **(B)** The number of H3K27me3-enriched loci that were up- or down-regulated more than 4 fold at any DAP is shown in box graphs. **(C)** Hierarchical cluster analysis of spatiotemporally regulated H3K27me3 target genes. **(D)** Venn diagrams to show the overlaps between H3K27me3-enriched loci and spatiotemporally regulated genes during endosperm development.

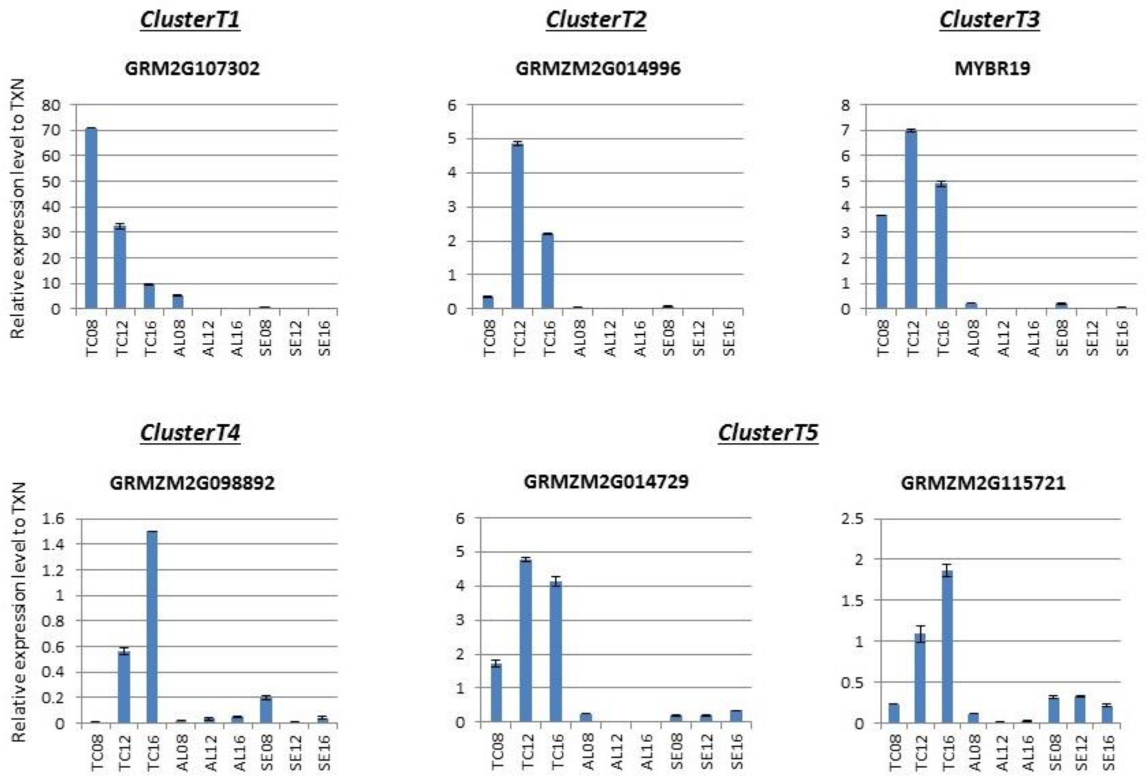

Figure S6. Validation of the expression of spatiotemporally regulated H3K27me3 target genes. Validation of the expression of spatiotemporally regulated H3K27me3 target genes. The six genes were tested using qRT-PCR analysis. *Y*-axis represents the relative expression level to *TXN* and *X*-axis indicates 9 samples: BETL08, BETL12, BETL16, AL08, AL12, AL16, SE08, SE12, and SE16 from left to right.

	BETL	AL	SE
H3K27me3 enrichment loci	0	0.001074	1.00E-06

	8 DAP	12 DAP	16 DAP
H3K27me3 enrichment loci	0.000186	0	0.000759

Figure S7. Statistical significance of the overlap between spatio-temporally regulated lncRNAs and H3K27me3-enriched loci. Chi-square test to address the significance of over-representation of H3K27me3-enriched loci in spatiotemporally regulated lncRNAs.

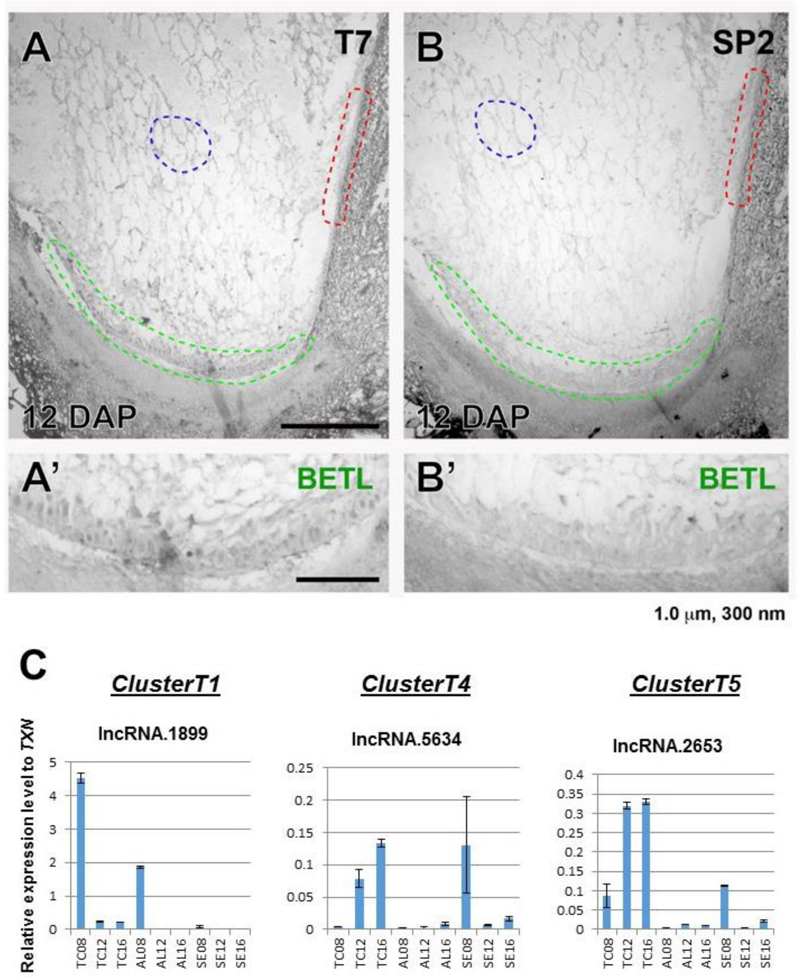

Figure S8. Validation of the expression of spatio-temporally regulated lncRNAs. (A) *in situ* hybridization of lncRNA.2653 (B)Three lncRNAs were tested using qRT-PCR analysis. *Y*-axis represents the relative expression level to *TXN* (GRMZM2G066612) and *X*-axis indicates 9 samples: BETL08, BETL12, BETL16, AL08, AL12, AL16, SE08, SE12, and SE16 from left to right.
